# Pb-Contaminated Soil from Quintero-Ventanas, Chile: Remediation Using *Sarcocornia neei*

**DOI:** 10.1155/2021/2974786

**Published:** 2021-02-23

**Authors:** Verónica Meza-Ramírez, Ximena Espinoza-Ortiz, Pamela Ramírez-Verdugo, Paulina Hernández-Lazcano, Paulina Rojas Hermosilla

**Affiliations:** ^1^Facultad de Ingeniería, Universidad de Playa Ancha, Av. Carvallo 270, Valparaíso 2490000, Chile; ^2^Herbario VALPL, Facultad de Ciencias Naturales y Exactas, Universidad de Playa Ancha, Valparaíso 2490000, Chile; ^3^Laboratorio de Histología Vegetal, Facultad de Ciencias Naturales y Exactas, Universidad de Playa Ancha, Valparaíso 2490000, Chile

## Abstract

A phytoremediation process for lead (Pb) under laboratory conditions on contaminated soil from the Puchuncaví commune, Valparaíso Province, Chile, was carried out. It analyzed the phytoremediation potential of *Sarcocornia neei* (Lag.) M.A. Alonso and M.B. Crespo. The plants were propagated beforehand and extracted from the El Yali wetland, a RAMSAR 878 site in Valparaíso. Soil fertility and heavy metal concentration analyses of composite samples were conducted, complying with established protocols and standard methodology for chemical and metal analyses. These analyses were conducted in the Soil Analysis Laboratory of the Pontificia Universidad Católica de Valparaíso. The aim was to analyse not only the tissue of plants from both areas but also the soil to identify the changes in different conditions in which the plants live. To determine the type of inferential analysis to be performed, a normality test was applied; however, it was deemed unsuitable, and therefore, the contrasts were developed using nonparametric tests, particularly Wilcoxon. R project software was used in the tests, especially the RCommander package, together with the Jamovi free-license statistical spreadsheet application. The analyses results of the soil samples indicated high concentrations of heavy metals, predominantly Pb at a concentration of 77.97 mg/kg, acidic soil indicated by pH between 5.77 and 6.38, low levels of electrical conductivity, and the presence of organic matter. A phytoremediation efficiency of 99% on soil samples was achieved. Preliminary results were compared against international regulations on the concentration of metals in soil. The histological sections showed that individual plants probably adapted to their environmental conditions.

## 1. Introduction

The rapid Chilean macroeconomic growth in the last 30 years has been supported mainly in the “mining boom” emerged from the neoliberal reforms imposed by the military dictatorship through the Foreign Inversion Statute (1974), the New Mining Concessions Law (1982), and Mining Code (1983) [1]. This process has been, as well as the main driver of a sustained growing in the energy consumption, which has resulted in the last 20 years, important in the increase of initiatives entries aimed to increase the installed power of the main interconnected electrical systems in Chile, the Norte Grande Interconnected System (SING) and the Central Interconnected System (SIC) [1].

Mining and smelting activities in a country undoubtedly promote job creation and benefit the national economy. However, mineral extraction and its related industries inevitably damage the natural texture and structure of the soil and lead to serious contamination from toxic environmental metals. Industrial activities are, therefore, associated with the contamination of environmental matrices, such as soil and water, as well as living beings [2–8].

Significant levels of heavy metal contamination occur in Puchuncaví, Quintero-Ventanas, where one of the most important industrial parks in Chile is located. The heavy metals in the soil pose a potential health risk to the population because of their characteristics of being nonbiodegradable, cumulative, toxic, and bioavailable [9].

Research by Guo et al. [10] has shown that humans living near contaminated areas and being exposed constantly to heavy metals could have substantial health risks. Results of meta-analyses by Chowdhury et al. [11] have indicated that exposure to As, Pb, Cd, and Cu is associated with an increased risk of cardiovascular and coronary heart disease. Such findings emphasise the association between toxic metals in the environment and an increased risk of cardiovascular disease beyond the functions of conventional behavioral risk factors.

However, it has been found that the ability of plants to accumulate heavy metals in their tissues can be used to control soil contamination. [12]. The practical initiatives of any kind for the remediation of the great surface of contaminated soils with Cu and other metals in the country are not yet known.  The level of research in this ambit only reaches on research that had been focused in the identification of species in sites with high concentration of Cu, with none having stablished, for the majority, if they are useful for the phytoextraction or other techniques of phytoremediation. These species include *Cenchrus echinatus*, *Erygerom berterianum*, *Mullinum spinokum*, *Nolana divaricata*, *Dactylium* sp., *Anthemis cotula*, *Baccharis salicifolia*, *Argemone subfusiformis*, *Oenothera picensis*, *Verbascum thapsus*, *Schinus polygama*, *Scirpus asper*, a*rmeria maritima*, *Trisetum lasiolepis*, *Montiopsis potentilloides*, and *Atriplex deserticola*. The majority of the species were identified in the nearing of the Fundicion Caletones, VI region, and in the vicinity of mines in Atacama region [13–16]. Another study carried out in Puchuncaví identifies several species capable of accumulating Cu, not referring to other metals, and the investigated species is *Oenothera affinis* which could be considered as a good candidate for remediation initiatives for Cu in Chile, since it is a native perennial herb, resistant to drought, and easy to propagate that produces a large biomass [17].

Accordingly, our research concerns the gathering and eventual provision of relevant information for future remediation of the soils of the Los Maitenes wetland, located in the Puchuncaví commune. In this research, we used the native species *Sarcocornia neei* (Lag.) M.A. Alonso and M.B. Crespo in applying phytoremediation techniques to soils contaminated with Pb, and we evaluated the decrease in the Pb concentrations in the soil samples after treatment.

## 2. Materials and Methods

### 2.1. Study Area

**Figure 1:**
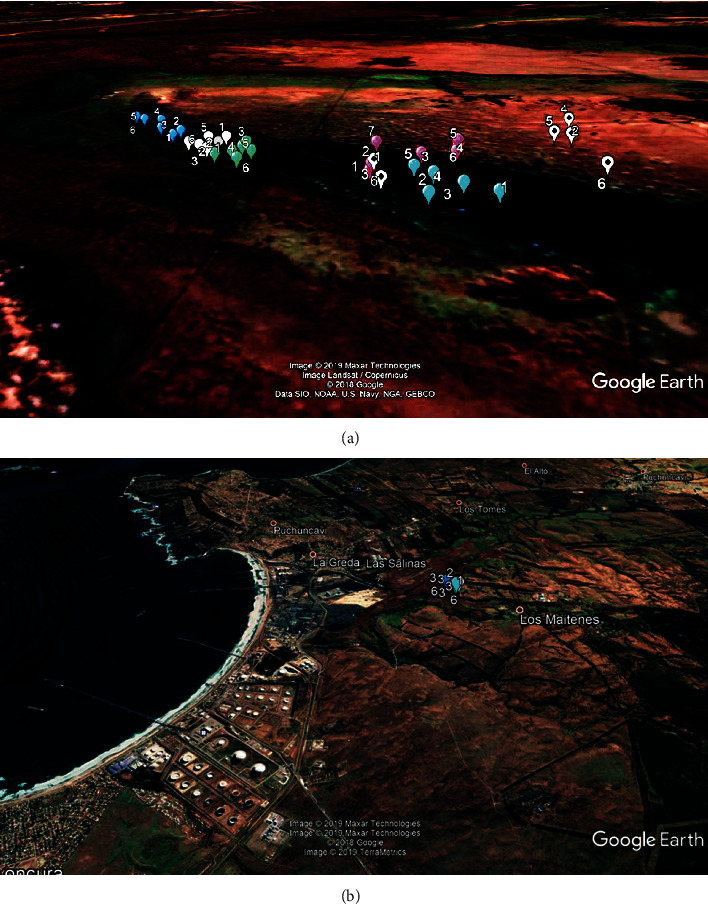
(a) Location of subsamples in Los Maitenes wetland. (b) Map showing the proximity to the industrial area.

Our research was conducted in two areas, namely, the Los Maitenes wetland and the El Yali wetland. We collected soil samples for phytoremediation from the Los Maitenes wetland, which is highly contaminated by heavy metals from industrial activities. The sampling points in the wetland are indicated in Figure 1. The second study area is the El Yali wetland, a natural reserve free of polluting activities, from where we collected specimens of the species *Sarcocornia neei*. The coordinates of the sampling areas in this wetland are indicated in Table 1.

### 2.2. Soil Sampling

We collected soil samples from six areas in the Los Maitenes wetland. Six subsamples, each weighing approximately 1.2 kg, were extracted randomly from the six sampling areas, complying with the established soil extraction protocols, Montenegro 2066, Nch 3400/2016 [18, 19]. The protocols suggest, e.g., that collection areas should be free of surface elements such as foreign substances and should be undisturbed areas. Sampling was performed by V-type cuts to prevent the earth from crumbling down the sides. In the first five zones, the cuts were between 10 cm and 20 cm deep, but in zone six, the depth was only between 5 cm and 15 cm because of the hardness of the soil.

The extracted subsamples were homogenised manually in a bucket to obtain a composite sample from each zone, breaking up soil lumps and removing residues such as roots or stones in the process [20]. Finally, the composite samples, each weighing approximately 7.22 kg, were stored in plastic bags and identified with I number de sample, zone, and mass.

For the analyses of heavy metal concentrations, soil fertility, and phytoremediation, each composite sample was presieved using a 2 mm sieve, as suggested in the literature [20]. Once the soil samples were prepared, 1 kg of soil composite sample from each zone was delivered to the Soil and Foliar Analysis Laboratory of the Pontificia Universidad Católica de Valparaíso. Fertility analysis followed the established methods [20]. The methodology used in the fertility parameters corresponds to methods of analysis recommended for Chile soils [20]. The methodology used in the fertility parameters correspond to recommended analysis methods for Chilean soils Zagal and Sadzawka (2006). For pH determination, Method 3.1 “Suspension and potentiometric determination” was used. For electrical conductivity (EC), Method 9.1 “1 : 5 Extract and determination by conductivimetry” was used. For the determination of organic carbon and organic matter (OM), Method 7.1 “Oxidation by dichromate in an acid medium and colorimetric determination of the reduced chromate” was used, in addition to Method 7.2 “Loss on calcination.” For the determination of nitrogen (N), Method 14.1 “Kjeldal digestion,” Method 14.1.1 “Distillation of NH_3_ and determination by titration,” and Method 14.2 “Extraction with potassium chloride 2 mol/L, distillation of NH_3_, and determination by degree” were used. To determine boron, Method 11.1 “Extraction with a solution of CaCl_2_ 0.01 mol/L at boiling and colorimetric determination with azomethine-H” and Method 11.2 “Extract of saturation and colorimetric determination with azomethine-H” were used. For the determination of calcium (Ca), magnesium (Mg), potassium (K), and sodium (Na), Method 4.1 “Extraction with ammonium acetate solution 1 mol/h at pH 7 and determination by absorption spectrophotometry and atomic emission, with lanthanum” was used. For the determination of phosphorus, Method 6.1 “Extraction with 0.5 mol/L sodium bicarbonate solution at pH 8.5 and colorimetric determination of molybdenum blue” and Method 6.2 “Phosphorus buffer capacity (CP)” were used. The methodology used for the determination of heavy metals corresponds to the “Protocol of methods of analysis for soils and sludge for Chile” Zagal and Sadzawka [21]. Methods 7.1 and 7.2 were used to perform soil digestions. Methods 8.1, 8.2, and 8.3 were used to perform atomic absorption spectrophotometry. Method 9.1.1 was used for the determination of arsenic in soils. Method 9.3.1 was used for the determination of zinc in soils. Method 9.4.1 was used for the determination of copper in soils. Method 9.7.1 was used for the determination of lead in soils. Method 9.5.1 was used for mercury. The equipment and brands used are specified as follows: atomic absorption spectroscopy with direct aspiration; the equipment is atomic absorption, Sensa AA Model, GBC Brand; the reagents used for absorption are of Merck Brand, and the purity is PA. For the metal mercury (Hg), standard EPA 3050/EPA 7471A was used for the more technical cold vapor digestion stage, digestion with sulfuric acid and nitric acid and potassium permanganate and potassium persulfate. Hydrúros generation equipment was used (Shimadzu and Shimadzu Atomic Absorption Spectrophotometer and Photron Mercury Hollow Cathode Lamp). The reagents used are hydrochloric acid (HCl), nitric acid (HNO_3_), sulfuric acid (H_2_SO_4_), KMnO_4_ potassium permanganate solution, sodium chloride solution (NaCl), and hydroxylamine ((NH_2_OH); brand: Merck; purity: PA. For atomic absorption spectrophotometry with direct aspiration, the equipment is the atomic absorption model Sensa AA, Brand GBC, and reactive used for the absorptions is MERCK, purity is PA. For the mercury metal (Hg), normative EPA 3050/EPA 7471A was used. For the digestion step with cold vapour technique, digestion with sulfuric acid and nitric acid, potassium permanganate, and potassium persulfate, SHIMADZU Hydrate Generator equipment and SHIMADZU Atomic Absorption Spectrophotometer were used. In mercury void cathode lamp PHOTRON, reactives used are hydrochloric acid (HCl), sulfuric acid (H_2_SO_4_), nitric acid (HNO_3_), potassium permanganate solution (KMnO_4_), sodium chloride (NaCl), and hydroxylamine (NH_2_OH), PA purity (MERCK).

### 2.3. Propagation

**Figure 2:**
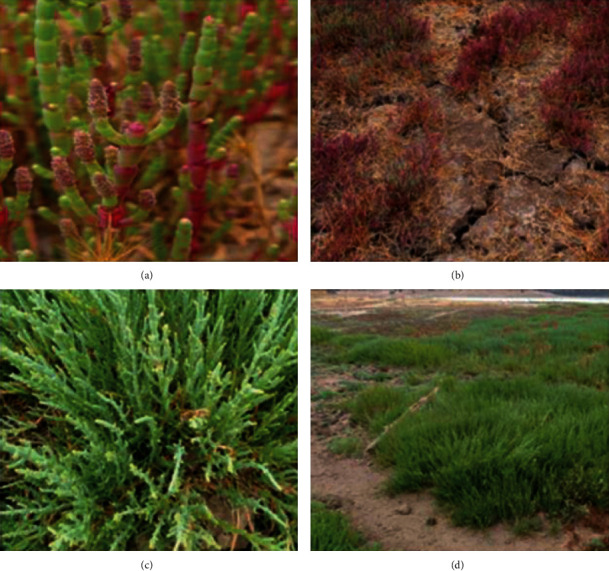
Status and morphology of *S. neei* in the coastal wetlands of Los Maitenes (a, b) and Matanzas (c, d).

**Figure 3:**
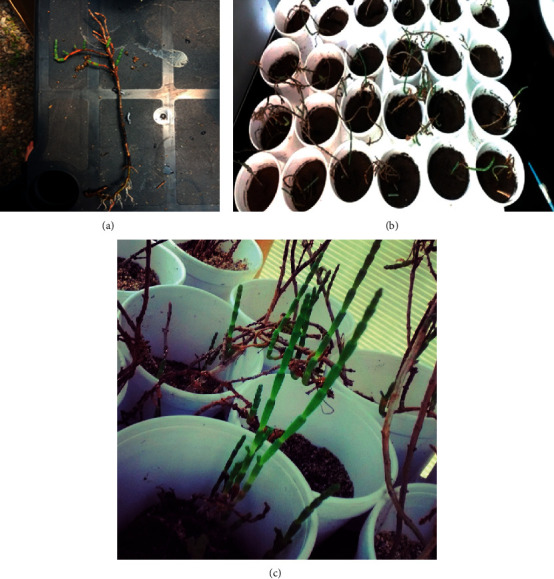
(a) Plant with root development in rhizome to be used in remediation. (b) Plants distributed in containers with contaminated soil. (c) Detail of growing plant with lateral budding beginning near the base.

We used a native plant species, *Sarcocornia neei*, native species from Chile and South America, considered a small shrub, which can be found erected or prostrated, sometimes crawling and rooting in knots, it height is from 80 to 150 cm, leaf tip rounded, and the inflorescence is 10–40 mm long and 3–4 mm wide (Alonso, M., and Crespo, M., 2008) [22]. Halophyte, succulent, is also a plant with  CAM type photosynthesis, which allows it, among other qualities, to resist high levels of salinity in its natural habitat. Figures 2 and 3 show specimens of different origin according to the presence of pollutants and plants propagated on contaminated soil.

To determine the development of the plants in soils with characteristics different from or similar to their habitat and to investigate their growth by various mechanisms, the plants were propagated in two types of soil from rhizomes and seedlings.

The plants collected from the El Yali wetland were placed in containers with water to preserve them for one week. As the *Sarcocornia neei* species develops in sandy soils, one of the substrates we used was sand collected at Playa Carvallo in Valparaíso because of the absence of industries in the zone and the proximity with the Facultad de Ingeniería Laboratories which is 300 m; this sand was sterilized for 105°C for 6 hours in a Pasteur stove. As the soil to be remediated is sandy clay, we used a mixed substrate consisting of sand and organic substrate or leaf soil Brand ANASAC, and garden was chosen because of pH characteristics 5.0–8.5, CE (dS/m) <3, MO (%) >20, humidity (%) 30 and 45, apparent density (kg/L) 0.4–0.7, relation C/N <50, and at a ratio of 50 : 50.

For propagation by rhizomes, we collected only young, healthy stems showing cutting growth and discarding dry, dehydrated, and thin stems. Subsequently, a uniform cut with three to six buds was made on each stem. A total of 43 rhizomes were immersed in water for one week, after which they were treated with a small proportion of gibberellic acid to promote rapid root formation. Subsequently, the specimens were planted vertically in a plastic container to prevent damage.

Propagation by seedlings initially followed the same procedure, with young plants of small size with healthy and large roots being selected. Subsequently, the plants were washed with distilled water to eliminate residues. Before being transplanted into pluvium containers, the 43 specimens used in this procedure were immersed in water for approximately one week, taking special care to avoid contact of the cuttings with water, as they have a tendency to rot with excess hydration.

### 2.4. Anatomic Description of *Sarcocornia neei*

To describe the plant tissues of *Sarcocornia neei*, samples of apices and roots of individual plants from the Yali National Reserve and from Puchuncaví were collected and fixed in FAA. Subsequently, they were dehydrated in a chain of ascending alcohols to be included later in paraffin, following the protocols of Bowles [23] and Montenegro [24]. This protocol allowed tissue cuts between 14 and 18 micron to be made in a microtome. The sections were stained with safranin, giving the nuclei and lignin of the secondary cell walls a reddish color, and with fast green, which stains primary walls and cytoplasm. The sheets were observed, analyzed, and photographed under a LEICA DMLB microscope (Leica Microsystems, Germany).

### 2.5. Statistics Analysis

To determine the type of inferential analysis to be conducted on the variables, we first carried out normality tests on the target variables, i.e., concentrations of Pb in the soil before and after the applied methodologies. In both instances, normality was rejected, and we opted to use a nonparametric data analysis method. The nonparametric tests corresponded to Wilcoxon tests for paired samples, as there was the same number of sample units in both instances, but the treatments differed. A statistical treatment was applied to the results, referring to the Pb concentrations obtained prior to the phytoremediation and after, and we used R software and the Jamovi spreadsheet application. These were chosen because they are low-code free software, according to the definition in the General Public License (GNU) of the Free Software Foundation (FSF). These applications are freely available for download [25, 26].

Employing these applications, we could determine the significance of the study, i.e., whether there were significant changes in experimentation after applying the phytoremediation methodology. We used the *p* value as a criterion based on the negation of the null hypothesis, which refers to the initial and final concentrations of Pb being the same, as demonstrated in the following expression:

(1) $$\begin{array}{c} H\_0:\mu \_A=\mu \_B, \end{array}$$

where *H*0 represents the nule hypothesis, *μA* represents the control group or initial concentration, and *μB* represents the experimental group or final concentration.

### 2.6. International Regulations

We compared our results against international regulations (Swedish, Canadian, Brazilian, Australian, and Spanish) pertaining to residential, industrial, and other land uses.

## 3. Results and Discussion

Analysis of samples from Los Maitenes wetland soil before remediation.

The results of the fertility analyses of the six soil samples collected from the Los Maitenes wetland are presented in Tables 2 and 3. The tables show an optimum range, which corresponds to an optimal range for average soil in which plants can develop. The results were obtained by the soil and foliar analysis laboratory, as mentioned before.

Based on the medium range for soil parameters or variables, our investigation indicated slightly acidic soils, with a low percentage of organic material (mainly in zones 4, 5, and 6), as well as electrical conductivity (EC) in all the zones, which is an indicator of soil quality [27].

As regards macronutrients, only potassium was indicated within the optimal development range for plants, whereas phosphorus levels were too high, and nitrogen was present at low concentrations (with the exception of zone 2). The availability of nitrogen is the main limiting factor in the productivity of plants and together with phosphorus determines plant growth [28].

The salts and minerals determined in the current analysis showed great variability in the high range compared with the middle range, which is unsuitable for adequate plant development. This indicates that the soil samples collected both lacked and exceeded optimal concentrations of essential elements; however, in the first instance, the results did not affect the phytoremediation of the soil with the native species *Sarcocornia neei*.

Table 4 shows the concentration results of heavy metals in soil samples collected from the Los Maitenes wetland, where the presence of Pb, As, and Hg was determined. These heavy metals are toxic to people, particularly to children and the elderly.

A comparison of the concentrations of the three metals clearly showed that Pb occurred in greater quantity, with the highest values detected in zones 1–3. This could be ascribed to there probably being contact with the water of the wetland in these areas, leading to sediment deposit being generated. Zone 6 showed a lower concentration of these metals, probably because of inadequate vegetation cover in this zone.

Previous analyses of the Los Maitenes wetland employing the same methodology have indicated Pb at high concentrations of 71.77 mg/kg and 81 mg/kg in 2014 and 2016, respectively [29].

The high metals concentration in the soils of Los Maitenes wetland is because of [30] the situation and location of Puchuncaví, and the industrial pole in the proximities has not changed in more than 30 years. The author indicates that it is striking that 30 years later, the same conclusions are still reached as in the two previous studies by Hermosilla and Rojas in 1988, where they used the spectrophotometric quantification of arsenic in hair and urine of people of different ages. The samples were collected in volunteers over 15 years of age and residents for at least 10 years in the study area or in a reference area (Valparaíso and Viña del Mar). The global analysis of the data demonstrates for urine, an average concentration of 0.042 ppm (range 0 to 0.309) in Puchuncaví v/s 0.024 ppm (range 0.005–0.110) in the control zone, and for hair, 2.178 ppm (range 0.013–18.023) v/s 0.434 ppm (range 0.015–1.526). Other authors, Cornejo and Romano in 1983, demonstrate high concentrations on cadmium, cooper manganese, lead, iron, zinc, arsenic, and sulphate ions in atmospheric sediment collected 1, 5, 10, 15, and 20 km from industrial complex, following the wind direction. The concentration of contaminants decrease with the distance of the source, and its geographic and temporal distribution can be explained because of topographic barriers and wind prevalence. An important factor was rain as well, which deposit the suspended elements in the air becoming a barrier to its dispersion. In the other hand, Tironi [31] points that atmospheric deposition in 2010 and 2011 in Puchuncaví locations was enriched with elements which include arsenic, cooper, lead, and zinc, among others and that contamination was higher in the proximities of the emission source. In clinical studies, the presence of lead and arsenic was established, which suggest chronical exposition to these metals, in the Escuela La Greda students, as well as the absence of known research in bioremediation.

The enrichment in the heavy metals concentration in the proximities of the Ventanas Industrial Complex and its effects on the biomagnification was studied by Puelles in the year 2018 [32] who analyzed biomagnification with arthropods; it was determined that those are considered toxic, such as As, Pb, and Cd, and are of concern. Zn and Cu are micronutrients, and therefore, there are mechanisms in living beings to regulate them; their biomagnification and bioaccumulation can be harmful to the terrestrial arthropod community. Of this group of metals, As and Pb showed a negative correlation with the general diversity of arthropods but not in the diversity by the functional group, where strong positive correlations were observed in richness and abundance, which can be explained by development tolerance to heavy metal contamination of the arthropod community of the place, either by acclimatization or adaptation, which translates into an increase in individuals of tolerant species. This poses a danger to the vertebrates that make up other levels of the terrestrial food webs, since they would consume the arthropods of the sector with large concentrations of heavy metals.

Studies related to native plant species in the Puchuncaví area indicate that there are species capable of accumulating Cu, in large quantities. These species are *Oenothera picensis* (614 mg/kg), *Baccharis linearis* (314 mg/kg), and *Argemone subfusiformis* (391 mg/kg). These species also produce large amounts of biomass, which makes them candidates for a phytoextraction process (González et al., 2008) [33].

### 3.1. Phytoremediation

The results of phytoremediation on the Los Maitenes wetland samples are presented in Table 5. These results indicated a considerable decrease in the samples from the six sampling zones, with the maximum value obtained being 1.47 mg/kg and the minimum 0 mg/kg.

Analyses of Tables 4–6 and Figure 2 relevant to the concentration of Pb before and after remediation indicated a significant decrease after treatment, with *p* < 0.05. This demonstrated the effectiveness of the remediation methodology on the contaminated soil samples in laboratory conditions. These results suggest that the remediation potential of the *Sarcocornia neei* plant should be investigated further. Our finding is consistent with the initial research results of Montenegro [24], which indicated absorption of metals from contaminated soil in the aerial structure of plants.

### 3.2. Statistical Analysis Using R Software

As a result of the Shapiro–Wilk normality test to specify the kind of inferential analysis to be used, the rejection of this is concluded, considering a significance level of 5% (Wilcoxon = 0.05, *p* value =  > 0.05). We used the Wilcoxon nonparametric test to determine whether there were significant differences between the mean concentration of Pb before and after remediation. The data supported evidence in favour of the alternative hypothesis, i.e., there were significant differences after both treatments. This obviously indicated that the concentration of Pb in the soil, measured in mg/kg, had been reduced (Wilcoxon = 0.031, *p* value = 0.001).

Investigation of other *Sarcocornia* species by Vårhammar et al. [34] showed that *Sarcocornia quinqueflora* absorbed Pb and other metals from contaminated estuaries. The contaminants accumulated mainly in the roots, with eventual translocation to the stems. Other species, such as Fabaceae and Poaceae, have been used after organic soil amendments (*Lolium multiflorum* Lam. Var. *italicum*, *Secale cereale* L., *Vicia villosa* Roth, and *Trifolium pratense* L.). The translocation of trace elements from root to shoot was low in all plants, indicating that the cultivation of the plants used in the study was safe with respect to the propagation of trace elements into the environment [34]. Several authors are in agreement with such statements, suggesting that metals are stored mainly in the root system and not the aerial parts, as metals are absorbed first by the roots, but they are translocated later to the aerial parts [35].

Likewise, in other studies with high Andean species [36], the concentration of heavy metals in the soil, root, and aerial part of the high Andean plant species exposed to treatment with soil from the polymetallic concentration of Mesapata, in greenhouse conditions where the order of highest concentration Pb > Zn > Cu > Cd > Ni was obtained, was categorised as phytoextractive plants *Stipa ichu* (Ruiz and Pav.) Kunth and *Pennisetum clandestinum* Hochst ex Chiov by obtaining a bioconcentration factor, BCF = 1.07, and a translocation factor, TF = 1.15, BCF = 1.62, and TF = 1.66, respectively.

Studies with increasing solutions of Cu concentrations in the species *Oenothera picensis* indicate that it is significantly found in aerial tissues and in the roots. The higher the concentration of copper in solution, the higher the concentration of copper in the aerial tissues and in the roots, in both populations [37, 38].

In our study, the correlation matrix, Table 7, shows a significant relationship between the heavy metals Cu, Pb, Fe, and As. This finding could be ascribed to the study area being close to an industrial zone, with foundries and thermoelectric plants probably emitting the heavy metals detected in the analyses. This positive correlation, albeit of smaller magnitude, corresponds with previous investigation results showing a significant correlation of Pb with Fe and Mn. In turn [38, 39], such correlation could be explained by Pb^2+^ followed by Cu^2+^ being adsorbed preferentially by the Fe and Mn oxides of the soil, which is favoured even more when the soil is alkaline.

As regards the high correlation between organic matter (OM) and metals, we suggest that this aspect requires further study because of the low levels found in the six composite samples.

### 3.3. Propagation by Roots

Forty-three specimens of *Sarcocornia neei* were planted in sand substrate and in a mixture of equal proportions with ANASAC brand organic substrate. All the specimens developed in the first week of propagation, with those planted in the mixed soil showing advanced development. This finding indicated that the ideal development environment for *Sarcocornia neei* is the sandy substrates, however, with enough clay to ensure adequate water retention. We found that this species needed constant watering every two to three days, in both roots and cuttings, as the plants tended to dehydrate when subjected to extremely high temperatures or direct sunlight.

This species is characterised by a tangled root formation that complicates the extraction of plants with their roots. However, extracting plants without roots could be a viable alternative for propagation, as development is achieved easily using rhizomes, the only disadvantage being the extended development time.

### 3.4. Propagation by Rhizomes

The specimens were planted in sand substrate, with 35 showing significant development (81.4%) at the second week of irrigation. Cuttings formation, stem growth and thickening, and root formation were observed.

As regards the remaining specimens, five presented much slower physical development and that only between weeks three and four, with some showing no stem development and only root development. Finally, only three specimens failed to spread. Copies were also planted in soil of transplantation with sand in the same proportion, where 43 copies were propagated. We observed rapid development of the plants after one to two weeks.

### 3.5. Anatomic Description of *Sarcocornia neei* (Lag.) M.A. Alonso and M.B. Crespo

*Sarcocornia neei* (Lag.) M.A. Alonso and M.B. Crespo is a perennial and halophyte plant between 80 and 150 cm tall, erect, or underinfested, with a rhizomatous stem. The plants sometimes show fragile adventitious roots at the base of the branches that are in contact with the substrate. Inflorescence of 10–40 × 3-4 mm occurs, and seeds are covered with hair. The plants generally inhabit saline soils and are distributed along the Pacific Coast from Peru to Chile, except in Estrecho de Magallanes [40].

**Figure 4:**
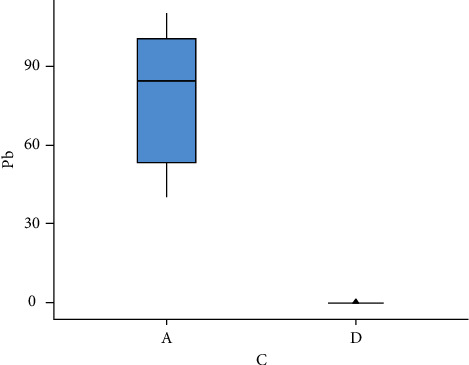
Graphic showing Pb concentration before and after phytoremediation. The [22] Jamovi project (2019) and [41] R Core Team (2018).

As regards the plant tissues of this species, the presence of several types of parenchyma stands out, which is ascribed to their habitat conditions, i.e., normally sandy and saline soils, wetland areas, and areas with flood and dry seasons. Individual plants generally have tissues similar to those presented by the plants from the El Yali National Reserve.  Figure 3 shows a cross-section of the apex, with phloem surrounding the central cylinder (FL), and another one forming islands, with the xylem between the tracheids. Although the aquifer parenchyma is not observed in the sample, it does exist outside the collenchyma, but it also has an aerifer parenchyma. This can be ascribed to the ability of the plants to adapt to different environmental conditions, which, in this case, are both humidity and drought that occur in the central part of Chile.  Figure 4 shows sections of specimens from a noncontaminated area.

**Figure 5:**
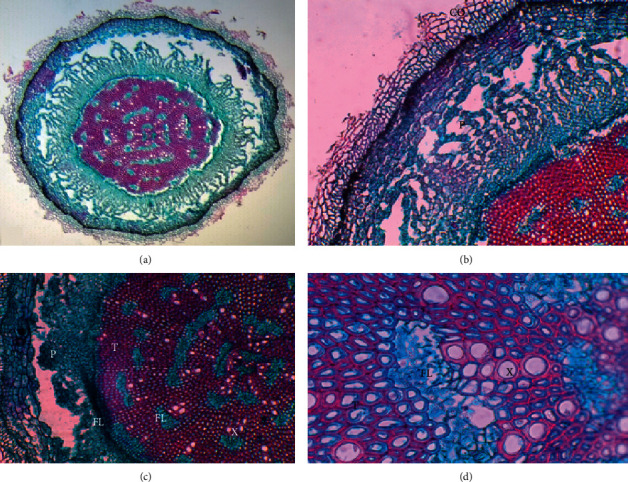
Image of apex cross section, El Yali National Reservation. Complete view of the stem, magnification 4X (a); collenchyma (CO), aerifer parenchyma (P), phloem (FL), and tracheids (T), 10X magnification (b); aerifer parenchyma (P), phloem (FL), tracheids (T), phloem (FL), and xylem (X), 10X magnification (c); tracheids (T), phloem (FL), and xylem (X), 40X magnification (d).

Figure 5, shows the cross-section of the stem extracted from a specimen sampled at Puchuncaví. It shows the aquifer, aerifer, and the medullary parenchyma that was not seen in the El Yali cut, as the histological cut was located at the apex of the stem, coinciding with the floral peduncle. As regards the xylem and phloem, they are grouped among the tracheids. This sample is quite similar in both sectors, i.e., no major differences were observed.

**Figure 6:**
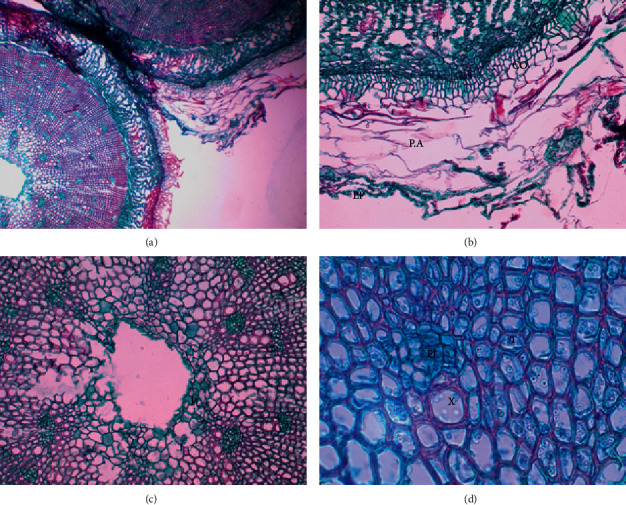
Image of cross-section of the stem from specimen from the Puchuncaví commune. Stem full view, 4X magnification (a); epidermis (EP), Aquifer parenchyma, collenchyma (CO), and parenchyma (P), 10X magnification (b); parenchyma (P), 10X magnification (c); vascular bundle (phloem and xylem) and tracheids (T) in 40X magnification (d).

**Figure 7:**
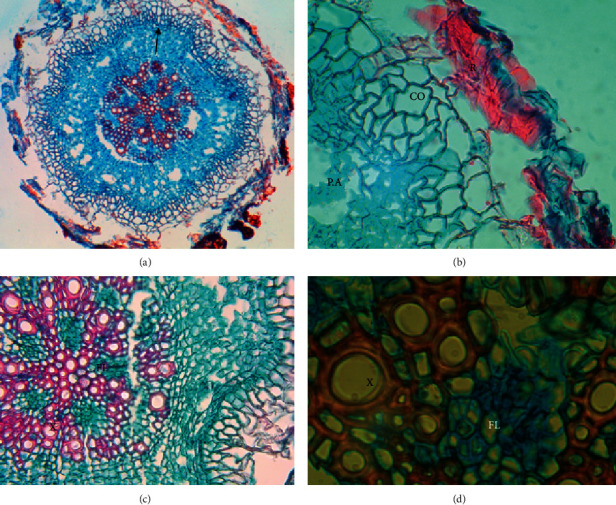
Image of root cross-section of specimen from El Yali National Reserve. General view of the root (a); rhizodermis (R), collenchyma (CO), and aeriferous parenchyma (PA), 10X magnification (b); phloem (FL) and xylem (X), 10X magnification (c); xylem (X) and phloem (FL) in 40X magnification (d) [42].

Figures 6 and 7 show the cross-sections of roots of the El Yali and Puchuncaví Reserve specimens, respectively. The El Yali specimen presents a typical root structure, with a relatively round cut with 5-6 xylem cords in the vascular bundle and collenchyma, and in this case, with two types of parenchyma and the aerifer being observed very well. In addition, the height of the xylem has radially repeating structures located under the collenchyma, which are probably cells that help transport water to the vascular cylinder.

**Figure 8:**
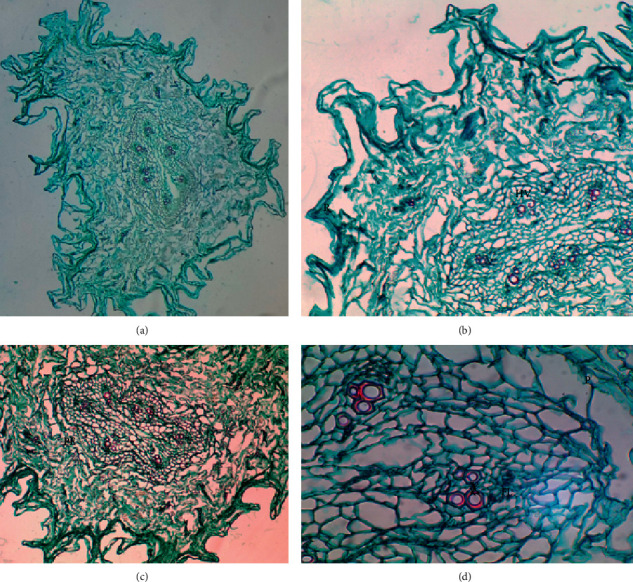
Image of root cross-section of the specimen from the Puchuncaví commune. Full root view, 4X magnification (a); rhizodermis (R) and vascular bundle (HV), 10X magnification (b); pericycle (P), 10X magnification (c); vascular bundle (phloem and xylem) in 40X magnification (d).

Figure 8 shows a quite particular root cut relevant to shape, irregular edge, deformed central cylinder, scattered parenchyma, and more widely spread vascular bundles. These characteristics can be ascribed probably to the plant adapting to the contamination conditions. The rhizodermis presents accumulations of some substances external to the plant.

### 3.6. Comparison against International Regulations

As no Chilean legislation or standards exist to regulate the determination of soil quality, international regulations are used to compare the levels of contamination present in the local soil. This is performed in accordance with Supreme Decree No. 40 of 2013 of the Ministry of Environment, which, in article 11, approves the Regulation of the Environmental Impact Assessment System. This regulation indicates the relevant reference standards that should be used, which are those in force in Germany, Argentina, Australia, Brazil, Canada, Spain, Mexico, United States of America, New Zealand, the Netherlands, Italy, Japan, Sweden, and Switzerland. In accordance with this decree, we selected regulations for soil quality assessment according to the different land uses in Canada, Australia, Brazil, Spain, and Sweden.

According to the regulatory plan of the Puchuncaví commune, the Los Maitenes wetland is not governed specifically for any particular land use. However, we compared our results with the specifications for rural, agricultural, and residential land use areas, as the soils of the Los Maitenes wetland show evidence of such activities in the environment. Figure 8 shows the regulatory limits in comparison with the soil quality of the study area before and after remediation.

**Figure 9:**
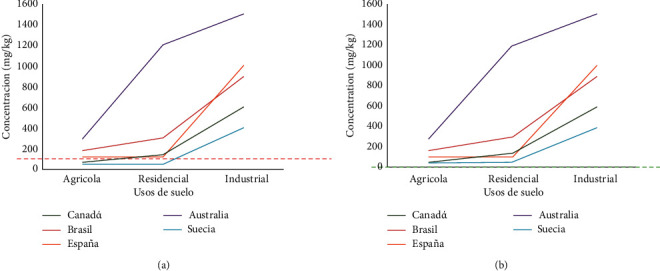
(a) Comparison of average Pb concentration with international regulations prior to phytoremediation. Own elaboration from [50] National Environment Center (2012). (b) Comparison of the average concentration of Pb with the international regulations after phytoremediation.

Comparison with the international regulations showed that both Canadian and Swedish standards were exceeded by the concentration levels detected in zones 1–3 and 4 (only the Swedish) from the Los Maitenes wetland relevant to agricultural use and sensitive soils (referring to agriculture, residential, and other uses). Therefore, although there are no suitable local regulations for such wetland soils, the international concentration level limits (Canada and Sweden) did indicate that the levels determined in our research pose a risk to the nearby population. However, as regards the Australian, Brazilian, and Spanish regulations, the land uses did comply with their standards. Figures 3 and 9 show the Pb concentrations before and after remediation in comparison with international standards.

It has been observed over time that succulent species have a significant capacity to accumulate elements and contaminants present in the soil. This capacity is reflected in our results for phytoremediation of the average soil samples with the *Sarcocornia neei* species, with 99.8% effectiveness being achieved for Pb remediation. The remediation percentage we obtained is significantly higher than that achieved in relevant other studies [43] and with Jara-Peña [44], obtaining 60% Pb in accumulator plants in the first year of treatment.

In view of the results we obtained, it can be inferred that according to the definitions in relevant legislation of the Environmental Ministry, Environmental Evaluation Service, and Environmental Superintendence, the soils in the Los Maitenes wetland of the Puchuncaví commune can be considered contaminated by heavy metals. Such contamination has caused the deterioration of the soil matrix of the ecosystem, which, in terms of the legislation, violates the right of citizens to live in an environment free of contamination and contravenes the act in terms of protection of the environment, as well as the preservation of nature and the environmental patrimony. These aspects are regulated by the provisions of the relevant legislation, without prejudice to what the legal norms establish on the matter and article no. 39 of the same legislation. This situation suggests that the Chilean government has not adequately watched over the conservation of the soil resource of the country.

However, with the remediation process we carried out, the Pb concentrations were reduced by 100%, demonstrating that it is possible to comply with the regulations chosen to carry out our analyses.

According to Malecka et al. [45], abiotic stresses, including metals in the soil, can be considered the main cause of the overall reduction (up to 70%) in vegetable yield and, therefore, a major limiting factor to crop production. This situation has deteriorated because of imbalances between crop production and population growth. Therefore, it is particularly important to understand the response of vegetation to such stressors, and as found in this research, the morphological changes at the root level can be appreciated.

For agricultural soil, the limit for the arsenic metal (As) acceptable is 20.0 ppm, according to the recommendation of European Community. In this investigation, the level of As found in soil was 62.4 mg/kg in zone 1 and 26.6 mm/kg in zone 6 with a mean of 45.2 mg/kg.

In the case of other metals determined in the soil of the contaminated place, it can be pointed out that the danger to the environment of As is demonstrated by investigations that indicate that it can be transmitted to the food chain through water to the soil and from this to agricultural crops, such that long-term use of arsenic-contaminated groundwater for irrigation may result in further increases in the concentration of arsenic in agricultural soil. [46]

Approximately 100 million people in more than 55 countries depend on artisanal small-scale mining, which produces between 20% and 30% of the world's gold. Between 10 and 15 million miners are involved in this work, of which 4.5 million are women and one million are children [47]

Heavy metals such as mercury (Hg) are a growing problem of environmental pollution worldwide. This can be found in soils naturally or due to anthropic activities, such as gold and copper exploitation. In Latin American countries, the amount of Hg released into the environment, in this type of mining activity, has been estimated between 80 and 100 tons per year. Once in the soil, this pollutant can transform into more toxic species, enter the trophic chain, and finally reach man and generate serious neurological and teratogenic problems [48]

Camargo Garcia et al. [49] conclude that the higher Hg contents in sites near the assemblies show the effect of mining activities on the soil resource in soil.

There is evidence for the difference in the regulations that regulate maximum concentrations; the average Hg values found did not exceed the threshold contents established for agricultural soils in other Latin American countries such as Mexico, but they did for Europe where the critical values are considered above 1 mg/kg. In this investigation, the levels determined were 9.26 mg/kg in zone 3 and 1.87 mg/kg in zone 6, with an average of 6.27 mg/kg.

Future aspects of the study considered evaluating the extraction of As, Hg, and Cu by the species *S. neei*, scaling up the investigation in small areas in areas of Puchuncaví and study the photosynthetic behavior of the species in contaminated areas and in areas free of contamination. Investigate in a grid or grid the accumulation of metals in the root, middle, and apex of the plant, as well as the rates of bioaccumulation and metal translocation.

In the long term, the research aims to study stressful techniques in the species in order to generate hyperaccumulating varieties in order to apply strategies that include affected communities in different areas of the country affected by metal contamination.

## 4. Conclusions

Our chemical analyzes indicated that the soil was slightly acidic, with the pH fluctuating between 5.77 and 6.38. In addition, we detected low EC, low OM levels (indicating poor soil fertility in all the sampling areas), and high concentrations of heavy metals (Hg, Pb, and As), highlighting above them the concentration of Pb.

Phytoremediation techniques were applied to the soil samples collected for the extraction of Pb, with a decrease of 99.8% in the Pb concentration being achieved, exceeded the prescribed limits for agricultural and sensitive land use.

After phytoremediation, a most significant decrease in Pb concentrations was observed, enabling compliance with the prescribed international limits.

Previous research has shown that the native species *Sarcocornia neei* has high potential for Pb remediation. Accordingly, using these plants in remediation could be a solution to the serious problem of heavy metal contamination in the Puchuncaví commune and, specifically, in the Los Maitenes wetland.

As regards to the observation of the histological sections, we concluded that the morphological changes could be ascribed to environmental influences, as such adjustments are necessary for plant survival. The presence of metals could not be affirmed with this methodology, although accumulations of substances were observed that should not be present in the rhizodermis, indicating these do exist.

## Figures and Tables

**Table 1:** Coordinates for extraction points of *Sarcocornia neei*, Yali wetland.

Site	1	2	3	4	5	6	7
—	19 H	19 H	19 H	19 H	19 H	19 H	19 H
Lat. S	33°45'11.24”	33°45'9.07”	33°45'6.42”	33°45'5.05”	33°45'5.42”	3°45'3.34”	33°45'3.16”S
Long. O	71°43'1.16”	71°42'59.50”	71°42'57.44”	71°42'56.15”	71°42'53.87”	71°42'51.32”	71°42'49.14”O

**Table 2:** Values of fertility analysis in samples from soil collected from Los Maitenes wetland.

Shows	pH at 25°C	Electric conductivity at 25°C (dS/m)	Organic material (%)	N (mg/kg)	Zn (mg/kg)	Mn (mg/kg)	Fe (mg/kg)
Optimum range	6.5–7.5	1.0–2.5	5.1–1.0	21–35	0.50–1.00	0.6–1.0	2.6–4.5
Zone 1	5.77	0.33	3.41	9.03	20.70	12.90	72.30
Zone 2	6.10	0.57	4.08	33.00	26.20	5.95	69.00
Zone 3	6.18	0.47	3.71	9.59	22.80	10.70	71.00
Zone 4	6.38	0.29	1.86	5.53	14.70	7.82	63.70
Zone 5	6.29	0.26	0.53	5.81	12.10	8.22	41.90
Zone 6	5.99	0.85	0.99	4.97	14.60	42.70	4590

**Table 3:** Values of fertility analysis from soil samples collected from Los Maitenes wetland.

Shows	B (mg/kg)	Cu (mg/kg)	P (mg/kg)	K (mg/kg)	K (cmol+/kg)	Na (cmol+/kg)	Ca (cmol + /kg)	Mn (cmol + /kg)
Optimum range	0.51–1.03	0.3–0.5	10.1–20.0	100–180	0.26–0.51	0.21–0.30	5.01–9.00	0.51–1.00
Zone 1	0.73	600.00	33.00	85.20	0.22	0.36	14.50	6.57
Zone 2	0.78	448.00	44.70	185.00	0.47	0.28	14.30	4.47
Zone 3	1.09	420.00	30.80	164.00	0.42	0.38	11.70	5.24
Zone 4	0.71	168.00	27.80	84.10	0.22	0.28	10.30	4.31
Zone 5	0.61	164.00	30.90	88.50	0.23	0.24	7.68	2.82
Zone 6	0.86	132.00	13.80	132.00	0.34	0.19	2.85	0.88

**Table 4:** Pb, As, and Hg concentrations in samples collected from Los Maitenes wetland soil.

Zones	Pb (mg/kg)	As (mg/kg)	Hg (mg/kg)
Zone 1	101	62.4	5.62
Zone 2	110	59.5	9.09
Zone 3	99.1	56.5	9.26
Zone 4	69.7	38.8	4.00
Zone 5	48.0	27.4	7.78
Zone 6	40.0	26.6	1.87

**Table 5:** Pb average concentrations determined after phytoremediation.

Concentrations (mg/kg)					
Zone 1	Zone 2	Zone 3	Zone 4	Zone 5	Zone 6
BDL	BDL	0.145	0.368	BDL	0.135

**Table 6:** Paired samples test. Pb before and after remediation.

Paired samples *T*-test					
—	—	—	Statistic	Df	*p*
Pb	Pb!	Student's *t*	6.41	5.00	0.001
—	—	Wilcoxon *W*	21.0	—	0.031

**Table 7:** Correlations matrix between soil variables.

Correlation matrix		pH at 25°C	Electric conductivity at 25°C (dS/m)	Organic material (%)	N (mg/kg)	Fe (mg/kg)	Cu (mg/kg)	Pb (mg/kg)	As (mg/kg)	Hg (mg/kg)
pH a 25°C	Pearson's r	—								
	*p* value	—								
Electric conductivity at 25°C (dS/m)	Pearson's r	−0.347	—							
	*p* value	0.500	—							
Organic material %	Pearson's r	−0.354	−0.011	—						
	*p* value	0.491	0.984	—						
N (mg/kg)	Pearson's r	−0.114	0.188	0.668	—					
	*p* value	0.829	0.721	0.147	—					
Fe (mg/kg)	Pearson's r	−0.280	−0.202	0.931^∗∗^	0.438	—				
	*p* value	0.590	0.701	0.007	0.385	—				
Cu (mg/kg)	Pearson's r	−0.642	−0.174	0.869^*∗*^	0.468	0.820^*∗*^	—			
	*p* value	0.169	0.742	0.024	0.349	0.046	—			
Pb (mg/kg)	Pearson's r	−0.299	−0.201	0.979^∗∗∗^	0.661	0.934^∗∗^	0.895^*∗*^	—		
	*p* value	0.564	0.703	<0.001	0.153	0.006	0.016	—		
As (mg/kg)	Pearson's r	−0.442	−0.173	0.970^∗∗^	0.573	0.941^∗∗^	0.951^∗∗^	0.984^∗∗∗^	—	
	*p* value	0.380	0.743	0.001	0.235	0.005	0.004	<0.001	—	
Hg (mg/kg)	Pearson's r	0.181	−0.364	0.564	0.563	0.386	0.492	0.633	0.546	—
	*p* value	0.731	0.479	0.244	0.245	0.449	0.322	0.178	0.262	—

The Jamovi project (2019); R Core Team (2018). ^∗^Significant association at 5%; ^∗∗^association significant at 0.5%; ^∗∗∗^association significant at 0.1%.

## Data Availability

The referenced specimens were entered into de VALPL herbarium of Universidad de Playa Ancha. VALPL-PLV 2267 voucher correspond to specimen from Ventana site and VALPL-PLV 2268 correspond to specimen from El Yali National Reserve. The data are available from the corresponding author upon request.
